# A cross-sectional study on the relationship between the risk of hypertension and obesity status among pre-adolescent girls from rural areas of Southeastern region of the United States

**DOI:** 10.1016/j.pmedr.2018.09.006

**Published:** 2018-09-13

**Authors:** E. Kipling Webster, Samuel W. Logan, Wendy N. Gray, Leah E. Robinson

**Affiliations:** a School of Kinesiology, Louisiana State University, Baton Rouge, LA, USA; b College of Biological and Population Health Sciences, Oregon State University, Corvallis, OR, USA; c Department of Psychology, Auburn University, Auburn, AL, USA; d School of Kinesiology, University of Michigan, Ann Arbor, MI, USA; e Center for Human Growth and Development, University of Michigan, Ann Arbor, MI, USA

**Keywords:** Hypertension, BMI, Obesity, Female

## Abstract

This study investigated early indications of hypertension risk and the association of overweight and obesity in young girls from a low socioeconomic region of the rural South. 139 females (*M* age = 8.85 ± 1.67 years) from a rural school in the Southeastern region of the United States served as participants. Body mass index was calculated based on the child's height and weight measurements (kg/m^2^) and resting blood pressure measurements were taken with calibrated, automatic oscillations devices. Girls who were overweight or obese were 2.81 times more likely to have a systolic blood pressure indicative of being at-risk/hypertensive (i.e., pre-hypertension and/or hypertension stage 1) than girls who were not overweight/obese. In fact, the percentage of overweight/obese girls who were at-risk/hypertensive was double that of girls who were not overweight/obese (43.2% versus 21.3%), respectively. Being overweight or obese is associated with almost three times a higher risk of hypertension than girls who are not overweight or obese.

In the United States, various social determinants such as socio-economic status, poverty, discrimination, residential segregation, and unequal access to health care greatly affect our health (Barr, 2014). Some of the greatest health disparities observed in the United States are in women of color (i.e., Black, Hispanic, and American Indian/Alaskan Native) (Barr, 2014). Diseases such as heart disease, stroke, diabetes, and cancer are among the leading causes of death for women of color (Barr, 2014; CDC, n.d.).

One factor that contributes to an increased risk of these diseases is weight and girls tend to be at a greater risk of being overweight or obese compared to boys, particularly as they approach puberty (Govindan et al., 2013). There has been a recent stabilization in the incidence of pediatric obesity in the United States (Ogden et al., 2014). However, historically, the prevalence of obesity and overweight children and youth has tripled over the past 30 years (Ogden et al., 2014). It is estimated that one out of three girls between the ages of 2–19 years are overweight or obese (Ogden et al., 2014). In Alabama, 35% of children are overweight or obese; placing the state among one of the ten states with the highest rates of childhood obesity (Robert Wood Johnson Foundation, n.d.).

Due to the fact that girls have a higher incidence of overweight and obesity, it is highly likely that these overweight and obese girls will become obese adults (Freedman et al., 2005), which places them at a higher risk for health complications that contribute to premature death (e.g., heart disease, type-2 diabetes, stroke, cancer, and hypertension) (Daniels, 2006). Hypertension, an adult-onset disease, is a condition where pressure in the arteries is elevated. Hypertension is rare in children, but there has been a rise in the prevalence in pediatric populations over the past two decades (Chorin et al., 2015; Freedman et al., 2012; Perng et al., 2016; Raitakari et al., 1994; Muntner et al., 2004; Robinson et al., 2013; Bao et al., 1995). Hypertension is a risk factor for coronary artery disease and an early warning sign for the development of atherosclerosis (Keramati et al., 2013; Luma and Spiotta, 2006). Recent studies have identified a clear link between weight and hypertension in children and youth (Friedmann et al., 2012; Hanevold et al., 2004; Angelopoulos et al., 2006; Ataei et al., 2009; Moore et al., 2006; Salvadori et al., 2008; Falkner et al., 2006). Children who are overweight or obese have higher values of systolic blood pressure (SBP) and diastolic blood pressure (DBP) compared to their normal weight peers (Friedmann et al., 2012; Falkner et al., 2006; Macdonald-Wallis et al., 2017; Sánchez-Zamorano et al., 2009; Hlaing et al., 2006). Additionally, these elevated values are maintained longitudinally (Juhola et al., 2011), highlighting the need for early detection for individuals at a greater risk for hypertension, like those who are overweight or obese.

There is a growing concern for obesity in United States girls, especially those exposed to negative social determinants (e.g., lower socio-economic status, lower-income communities, communities with mostly racial minority populations, states in the South and Midwest, and populations with lower education levels) that places them at a greater risk of long-term health complications (Barr, 2014). This need supports investigations to explore risk factors that are associated with increased weight status, particularly in diseases that until recently have been primarily observed in adults. This knowledge will help inform researchers and clinicians as they intervene and provide health behavior and educational programs that could aid in reducing the long-term health complications associated with hypertension. National data support that this concern and health disparities might be a greater problem in Southern and rural populations (Freedman et al., 2012; Robinson et al., 2013).

This study aimed to investigate early indications of hypertension risk and the association of overweight and obesity in children who were primarily from ethnic minority backgrounds living in a low socioeconomic region of the rural South. We hypothesized that youth who are overweight and obese would be at greater risk of having a hypertensive classification than those who are not overweight or obese. To further illustrate the impact of weight status on risk of having a hypertensive classification, we present exploratory analyses indicating the probability of a nine-year-old female (approximate mean age of our sample) having a hypertensive classification when their body mass index (BMI) is at 50th, 85th, 95th, and 99th percentile.

## 1 Methods

For this cross-sectional study, 139 female children and pre-adolescents (*M* age = 8.85 ± 1.67 years) from a rural school (i.e., Kindergarten through 6th grade) in the Southeastern region of the United States served as participants. African-American/Black children comprised the largest portion (82%) of the sample (Table 1). The median household income in the school district was $30,000 USD and all participants qualified for free or reduced lunch. The project protocol was approved by the Institutional Review Board. Before data collection, written informed consent from the parent/legal guardian and verbal assent from the participant were obtained. The exclusion criteria for the study was that no participant could already be on anti-hypertensive medication. This was confirmed with the school nurse and during the consent process. No participants were excluded from the study.

**Table 1:** Participant characteristics.

	Mean ± SD		
	Total sample	Normal weight	Overweight/obese
N	139	94	45
Age	8.85 ± 1.67	8.71 ± 1.65	9.13 ± 1.70
Race/ethnicity			
White/Caucasian	5.8%	6.38%	4.44%
Black/African-American	82.0%	80.85%	84.44%
Hispanic	11.5%	12.77%	8.89%
Bi-racial/multi-racial	0.7%	0.00%	2.22%
Systolic blood pressure category			
Normal	71.3%	78.72%	55.56%
Pre-hypertension	12.9%	9.57%	20.00%
Hypertension Stage 1	15.2%	11.70%	22.22%
Hypertension Stage 2	0.0%	0.00%	0.00%
Missing Data (n = 1)	0.7%	0.00%	2.22%
Diastolic blood pressure category			
Normal	79.9%	87.23%	64.44%
Pre-hypertension	10.1%	7.45%	15.56%
Hypertension Stage 1	7.9%	3.19%	17.78%
Hypertension Stage 2	2.2%	2.13%	2.22%

### 1.1 Demographic/anthropometric measures

Sex, date of birth, and racial/ethnic classifications were collected from the school records and parents/legal guardians. Height and weight measurements were taken during the school day by trained research assistants. Digital medical scales were used to measure height to the nearest 0.1 cm (Seca Stadiometer 220; SECA Corp. Hanover, MD) and weight to the nearest 0.1 kg (Seca Floor Scale 769, SECA Corp. Hanover, MD). Shoes and heavy outerwear were removed for both measurements and children were instructed to stand in a relaxed position with arms hanging freely to the side.

BMI was calculated based on the child's height and weight measurements (kg/m^2^) and compared to the Centers for Disease Control and Prevention sex-specific BMI-for-age normative growth charts to determine BMI percentile (Kuczmarski et al., 2002). These growth charts provide a representative sample to determine BMI percentile based on a child's age and sex. From these growth charts, children are classified as overweight if their BMI percentile is determined to be equal to or above the 85th percentile and obese if equal to or above the 95th percentile.

### 1.2 Hypertension

Resting blood pressure was used to indicate hypertension classification. Resting blood pressure measurements were taken by two trained research assistants with calibrated, automatic blood pressure monitors (Omron HEM-711 DLX, Omron Healthcare, Inc., Vernon Hill, IL) using a cuff size appropriate for each participant's upper arm size. Auscultation is considered the gold standard for resting blood pressure readings (USDHHS, 2005). But results from an automated oscillometric devices (AOD) are highly correlated with auscultation readings when calibrated and observer error and bias are minimized (USDHHS, 2005). Readings were taken while the participant was in a relaxed and seated position, feet flat on the floor, and arm resting at heart level with their palm facing upward. The right arm was used for all measurements.

Three measurements were taken from each participant. Before taking resting blood pressure, participants were seated in a private area. During this time, participants engaged in a relaxing activity for 5 min that included looking at magazines or coloring. After the relaxing activity, blood pressure measurements were taken with a one-minute rest period between each reading. This one-minute rest period was in accordance with established guidelines by the National High Blood Pressure Education Program Working Group (Falkner et al., 2004) on High Blood Pressure in Children and Adolescents. Based on previous work, resting blood pressure was reassessed one week later if there were any outliers (i.e., ≤ 10 mmHg difference in the final two readings or SBP was ≤120 mmHg) (Robinson et al., 2013).

For data analysis, the final two SBP and DBP readings were averaged to determine SBP and DBP percentile and classification using the United States Department of Health and Human Services (USDHHS) (2005) blood pressure classification tables. These tables take into consideration a child's sex, age, and height to classify, based on a representative sample, a child's SBP and DBP percentile (USDHHS, 2005). SBP and DBP classification are based on the following percentiles: 90th to the 95th percentile (prehypertension); 95th percentile to 5 mmHg above the 99th percentile (Stage 1 hypertension); equal to or >5 mmHg above the 99th percentile (Stage 2 hypertension) (USDHHS, 2005).

### 1.3 Data analytic plan

Descriptive statistics were calculated to characterize the sample. Pearson product (for interval scale data) and Spearman rho (for ordinal data) correlations examined the relations between BMI percentile and SBP and DBP readings. A chi-square analysis was conducted to examine the hypothesis that a greater number of participants who were overweight/obese would be classified as at-risk/hypertensive than participants who were not overweight/obese. An odds ratio was calculated from these data to determine the increased odds of being at-risk/hypertensive when overweight/obese. A follow-up exploratory logistic regression examined the combined role of BMI percentile and age predicting the presence of at-risk or hypertensive classification. Age was entered into the model as a proxy for duration of overweight/obesity, with the belief that the risk of having hypertension would be higher for those who were overweight/obese. As age was an imperfect estimate of duration of overweight/obesity, these analyses were deemed exploratory. A Receiver Operating Characteristic Curve (ROC) was created from the model data to optimize the predictive power of our model. All analyses were conducted in SPSS, version 23.0.

## 2 Results

### 2.1 Participant characteristics

Consistent with current prevalence rates of pediatric obesity (Ogden et al., 2014), one-third of the sample was overweight or obese. Twenty-eight percent of participants had an SBP that classified them above the normal range. That is, they are identified as pre-hypertensive, hypertensive stage 1, or hypertensive stage 2. No participants were classified as hypertensive stage 2 based on SBP. One-fifth (20.2%) of participants had a DBP above the normal range and 2.2% were classified as hypertensive stage 2 (Table 1).

### 2.2 Correlations among variables of interest

Correlations between BMI percentile, average SBP and DBP, and hypertensive classification (based on SBP and DBP) are presented in Table 2. As expected, higher BMI percentiles were moderately associated with higher SBP and DBP values and classification (*r* range = 0.30–0.45).

**Table 2:** Associations between BMI percentile, blood pressure, and blood pressure classification.

	1	2	3	4
1. BMI percentile	–			
2. Average systolic BP	0.45⁎	–		
3. Average diastolic BP	0.42⁎	0.69⁎	–	
4. Systolic BP classification	0.29⁎⁎	0.69⁎⁎	0.36⁎⁎	–
5. Diastolic BP classification	0.18⁎	0.45⁎⁎	0.69⁎⁎	0.41⁎⁎

⁎ Pearson correlation is significant at the *p* < .01 level.

⁎⁎ Pearson correlation is significant at the *p* < .001 level.

### 2.3 BMI category predicting hypertensive classification

Due to the small number of participants with a BMI value in the overweight category (i.e., 10.1%), overweight and obese categories were combined to examine the relationship between BMI category and hypertension classification. There was a significant association between a participant's BMI category and whether or not they were at-risk/hypertensive according to SBP, *X*^2^(1, N = 138) = 7.09, *p* < .01. Girls who were overweight or obese were 2.81 times more likely to have an SBP indicative of being at-risk/hypertensive (i.e., pre-hypertension and/or hypertension stage 1) than girls who were not overweight/obese. In fact, the percentage of overweight/obese girls who were at-risk/hypertensive was double that of girls who were not overweight/obese (43.2% versus 21.3%), respectively.

### 2.4 BMI and age predicting hypertensive classification

An exploratory analysis was conducted to examine the combined predictive power of age and BMI percentile correctly classifying participants as normal or at-risk/hypertensive. The initial model was significant, *X*^2^(2, N = 138) = 18.57, *p* < .001 (Table 3). For every percentile point increase in BMI, there was a two percentage point increase in risk for the presence of at-risk/hypertensive classification (OR = 1.02, 95% CI = 1.01–1.04). Age was not a significant predictor (*p* = .058).

**Table 3:** BMI percentile and age predicting systolic blood pressure classification.

Predictor	B	SE B	e^B^	95% CI
Age	0.23	0.12	1.26	0.99–1.60
BMI percentile	0.02	0.01	1.02	1.01–1.04
Constant	−4.40	1.18	0.01	

Based on information obtained from the ROC curve analysis, the optimal sensitivity and specificity of our model occur with a cut-point of 0.29. Using this cut-point, our model correctly identifies the presence of at-risk/hypertensive classification 77% of the time (sensitivity) and correctly identifies the absence of at-risk/hypertensive classification 68% of the time (specificity).

According to our model, the probability of a nine year-old female (approximate mean age of our sample) being at-risk/hypertensive is 22.59% with a BMI at the 50th percentile. This increases to 38.30% with a BMI at the 85th percentile, 43.51% at the 95th percentile, and 45.64% at the 99th percentile.

## 3 Discussion

Our results indicate that a higher BMI is associated with a greater risk of hypertension for girls located in a rural, low socioeconomic region of the Southeastern US. Our results also suggest that the probability of a hypertension diagnosis increases as BMI values transition from normal-weight to overweight to obese classification. Although recent research indicates that pediatric obesity rates have plateaued (Ogden et al., 2014), it is still disturbing that nearly 1/3 of the young, female participants in this study, were classified as overweight or obese. There are potential long-term health implications for overweight and obese children who develop and maintain hypertension during and throughout childhood years. Over time, sustained hypertension becomes a significant risk factor for several serious health conditions such as stroke and heart disease (Keramati et al., 2013; Luma and Spiotta, 2006).

Regarding clinical implications, our study demonstrated two startling findings. First, overweight or obese girls from a rural, low socioeconomic area are almost three times more likely to be at-risk of hypertension (i.e., prehypertension or hypertension stage 1) than girls who are not overweight or obese. Second, the percentage of overweight/obese girls who were at-risk/hypertensive was double the percentage for girls who were not overweight/obese (43.2% versus 21.3%). Hypertension affects about 70 million (29%) American adults (Nwankwo et al., 2013), and it increases one's risk of heart disease and stroke. Hypertension is also a huge financial burden to our health care costs. In 2011, the estimated total costs associated with hypertension in adults living in the United States were $46 billion dollars (Mozaffarian et al., 2015). These costs include but are not limited to: health care services, medications, and missed days of work. Gilmer and colleagues recently examined the impact of hypertension on health care cost in over 70,000 children between the ages of 3–17 years (Gilmer et al., 2014). When adjusting for BMI, children with normal blood pressure average annual health care per year was $736 while those classified with pre-hypertension or hypertension averaged $945/year and $1972/year, respectively.

Our current findings, along with previous research, highlight the current public health crisis on childhood overweight and obesity in the United States and the need to address the problem of childhood obesity. It is important for researchers to continue to understand the risk factors associated with the development of overweight and obesity in pediatric populations. The results draw attention to the importance of BMI screening in young children; particularly since our findings indicate that a higher BMI is associated with other health complications.

There are many contributing factors to the obesity epidemic including poor nutrition and dietary choices (Batch and Baur, 2005), lack of access to healthy food options (Ghosh-Dastidar et al., 2014), lack of physical activity (Sallis et al., 2012), family medical history (Ahmad et al., 2013), among others. Previous work has shown that BMI was a greater longitudinal predictor for higher blood pressure readings than physical activity or sedentary behavior in similarly aged children. This relationship was stronger among girls (Macdonald-Wallis et al., 2017). Indeed, our findings supports the need for early screening and identification of all potential risk factors to hypertension, especially in a rural and female population. A unique aspect of the study, even though it was not the purpose nor explored, was the SES of the children. All of the participants qualified for the free or reduced lunch and the overall median household income was $30,000 USD. Thus, at an early age, social class (i.e., SES) is another determinant that adversely affects children's health. One of the strengths of this study is the use of readily available, easy to use methods for measuring resting blood pressure and weight status. The measurement tools allow early childhood and school practitioners and health care providers the ability to measure and screen for hypertension as a preventive measure. This initial screening could lead to effective early interventions and educational programs aimed to decrease unhealthy weight status and prevent individuals from developing hypertension. Although the development of hypertension is the result of several social determinants, unhealthy weight is one of the most significant contributing factors. It is important to continue to understand the many aspects of childhood obesity and to explore the most effective intervention and education strategies toward the prevention of diseases at an early age and to promote positive health outcomes of children.

There were limitations to the present study. One limitation is the use of an AOD to measure resting blood pressure, as the use of AODs could lead to misclassification of prehypertension or hypertension due to overestimation (Flynn et al., 2012). However, AODs are highly correlated with auscultation readings (i.e., the ‘gold standard’), when calibrated, as we did in this study. The calibration and standardized procedures using the AODs minimizes measurement error and bias (USDHHS, 2005). Formal assessment of resting blood pressure through auscultation by professional health clinicians such as doctors and/or nurses in clinical settings may induce a higher blood pressure reading due to the “white coat effect” or the increased anxiety of individuals (Franklin et al., 2013). For this study, we created an informal and relaxed setting for participants before the collection of resting blood pressure. Participants were familiar with all of the researchers who have a long-standing relationship with the faculty, staff, and students at the school. This was a cross-sectional study where children were measured on one occasion, causal relationships and diagnostic classification cannot be determined. Therefore, caution is advised when examining and interpreting this data; multiple visits where elevated BP is detected is needed before diagnosing a child with hypertension. In the present study, potential confounding variables that may contribute to hypertension were not examined such as pubertal status which has shown to be associated with elevated SBP levels (Shankar et al., 2005), diet, physical activity behaviors, or family medical history. Future work is needed to understand the impact of multiple factors that may be associated with higher blood pressure in girls.

Another limitation is the use of BMI to classify participants as overweight or obese. This is a commonly used method in research and is appropriate for initial screening, but there are limitations associated with BMI as an index for weight classification (Nevill et al., 2006). Specifically, BMI does not provide an understanding of body composition or body fat percentage. Additional factors that may contribute to a child being overweight/obese as well as common comorbidities of obesity (e.g., Type 2 diabetes) were not examined. Factors such as physical activity levels, diet, family medical history, and sleep disordered breathing may play a role in increasing SBP or DBP independent of the impact obesity has on children. The present study was only focused on the impact of obesity, which relates to the practical study design and implications targeted, however this may limit the understanding of how obesogenic factors contribute to higher level of blood pressure in young girls. The demographics of our sample (i.e., primarily African American located in a rural, low socioeconomic region of the Southeastern US) limit the generalizability of our findings to the broader childhood obesity population. However, because African American youth are underrepresented in the research literature, yet are disproportionately at greater risk for obesity compared to Caucasian/White youth, our emphasis on this at-risk population is a strength.

## 4 Conclusion

There is a need for investigations to explore risk factors that are associated with increased weight status, particularly in diseases that until recently have been primarily observed in adults. This study aimed to investigate early indications of hypertension risk and the association of overweight and obesity in young girls from a low socioeconomic region of the rural South. One-third of young girls in the current study were overweight and obese; the increased risk of hypertension associated with increased weight status is a concerning trend in early childhood. Interventions should be investigated to target and reduce childhood obesity, as well as aimed to lower the observed heightened levels of blood pressure which have been shown to track into adulthood.
